# The Role of Campus Food Pantries in the Food Security Safety Net: On-Going or Emergency Use at a Midwest Campus Pantry

**DOI:** 10.3390/nu14224876

**Published:** 2022-11-18

**Authors:** Ana Mitchell, Melissa Pflugh Prescott

**Affiliations:** 1 Division of Nutritional Sciences, University of Illinois at Urbana-Champaign, Urbana, IL 61801, USA; 2 Department of Food Science and Human Nutrition, University of Illinois at Urbana-Champaign, Urbana, IL 61801, USA

**Keywords:** food insecurity, food pantry, college, students, satisfaction, acceptability, reach

## Abstract

Food pantries are an integral part of the food security safety net and were designed to distribute emergency food to alleviate short-term hunger. Given many rely on assistance long-term, food pantries may no longer meet the nutritional needs of the typical food pantry user. Less is known about the extent college students use campus food pantries and whether they seek ongoing food support. A comprehensive analysis of food pantry use, including reach, awareness, and student satisfaction was conducted using a cross-sectional campus survey, a student satisfaction survey, and observational data from pantry logs collected from August 2020 to May 2022. During the first year of operation, 20.6% of students were aware of the pantry, 3.1% of students were reached, and student satisfaction was high. About half of users visited once, while 15.4% visited 8 or more times during an academic year. On average, students that visited more had a larger span of use (6.5 months), visited more frequently (2 weeks between visits), and were more likely to be graduate students and older. While most students used the pantry in the short-term, chronic use of the pantry increased from year 1 to year 2. This suggests need may be growing and additional policies or programs are warranted to support students with chronic food needs.

## 1. Introduction

Food insecurity largely affects students historically underrepresented in higher education and hinders efforts to increase college degree attainment to economically diverse populations [1]. Food insecurity during college is associated with worse health outcomes [2] and factors that compromise student success, including lower graduation rates and educational attainment [3]. The pressing issue of campus food insecurity has been met with calls to implement systems-level and campus-level policies and programs [4]. Currently, campus food pantries are the primary means by which postsecondary institutions are addressing food insecurity [1]. Campus food pantries are now widely implemented but remain understudied [5]. Campus food pantries will likely continue to be implemented and/or sustained on college campuses until campus food security improves, or until other solutions that improve college food security are developed and widely implemented [6].

Food pantries were originally intended to help individuals gain short-term, emergency access to food [6,7,8,9]; however, food pantries, including campus food pantries, appear to be providing long-term support [6,7,8,10,11]. Therefore, the initial mission of food pantries may no longer meet the nutritional needs of the typical food pantry user [9]. This change in use—from emergency to continuous—may have contributed to the controversial debate about the role of food pantries in the food security safety net [12,13]. The food security safety net is comprised of programs that help those experiencing livelihood shocks and stressors acquire food [14,15]. The goal of the food security safety net is to help individuals escape food insecurity and hunger and achieve healthful, nutritious diets [14,15]. Like food pantries in other settings, campus food pantries may not be used for emergency, short-term food assistance. Students may utilize campus food pantries for ongoing support if they experience persistent food insecurity or if food pantries are the only food assistance available. More research is needed to understand whether campus pantries are being relied upon to acquire food in the short-term or long-term.

Previous research on campus food pantries has largely focused on basic information concerning student use. Studies have explored: awareness and reach [16,17], student user demographics [16,18,19,20,21,22], average student visits [6,21], reasons that students seek support [21], and obstacles that inhibit student use [16,23,24]. Studies on awareness and reach found student awareness ranges from 28.7% to 85.6% and reach ranges from 7.7% to 15.6% [16,17]. For food insecure students, reach ranges from 22.2% to 38.5% [16,17]. The demographic characteristics of pantry users vary largely by campus [16,18,19,20,21,22]. Barriers to utilizing campus food pantries have been well-documented and largely include stigma and students feeling that the resource is for those in more dire circumstances [16,23,24]. Pantry use has primarily been reported as the average number of visits per month or year. Pantry use has also been reported as the percentage of one-time users versus recurrent users. One study found student users visited 3.66 times per month on average [25], while another study found students visited 3.22 times on average per year [21]. Two studies reported about half (51% to 55.7%) of all food pantry users visit more than once per year [20,21]. Most of the studies evaluating campus food pantries are cross-sectional and consist of self-reported data on pantry use. Less is known about how students use campus food pantries over the course of the academic year and the extent of support sought.

Only one study has used observational data to assess food pantry use over the course of multiple years [22]. In this study, the number of students utilizing a mid-sized, upper Midwest campus food pantry grew each year and, there was an increasing proportion of students that used the pantry more than once a month during each year of operation [22]. About 15.1% of student users visited “regularly,” defined as visiting 8 or more times during an academic year, however, in this study, use was restricted to twice a month [22]. Therefore, less is known about the use of campus food pantries that are unrestricted, and more comprehensive studies on student use are needed.

Beyond student use, understanding how pantries are received by students is an important component to inform future implementation. Validating student satisfaction is necessary to establish better policies and practices [21]. Without assessing it, one might mistakenly equate continued use with student satisfaction when chronic use may be driven by sustained need and overshadow the opportunity to improve pantry implementation. To date, only one study has evaluated student satisfaction with campus food pantries. The study reported that students were highly satisfied, and qualitative responses were leveraged to make program improvements like extending hours of operation and providing larger amounts of fresh produce and canned or frozen meats [21].

This paper addresses the gap in student satisfaction with campus food pantries and the extent to which students use unrestricted pantries over the course of the academic year. The specific aims of the present study were to (1) assess awareness, reach, and use of a campus food pantry by food security status, (2) examine patterns of student use over the course of two academic years, and (3) determine student satisfaction and acceptability of the campus food pantry.

## 2. Materials and Methods

### 2.1. Program and Link2Feed Data

A food pantry named the “Food Assistance & Well-being Program” opened at the campus recreation center of a large Midwestern public university in August 2020. The pantry was established as a satellite location to a preexisting community pantry. The goal was to provide acute supplemental food assistance to students in need and to ensure access to nutritious food. The pantry was open twice a week (Tuesday 1–4 pm and Saturday 2–5 pm) and students could visit as needed without restriction. To use the pantry, students had to first enter the recreation center using a valid student ID, and then upon arriving at the pantry, students created a profile with an online program, Link2Feed. At each visit, students signed into their account and after each opening, pantry staff downloaded the log of student users without identifiers. Each account includes a unique identification number and student sociodemographic characteristics: date of birth, gender, marital status, housing type, ethnicity, members of their household, education level, employment, and whether individuals self-identify as being impacted by coronavirus-19 (COVID-19), or whether they have a disability, veteran status, or other. The data from the first two years of implementation (August 2020–May 2021 and August 2021–May 2022) was shared with the research team. Prior to the pantry opening, the study protocol was deemed exempt by the Institutional Review Board at the University of Illinois at Urbana-Champaign (IRB #21148).

### 2.2. Campus Survey

Data from a campus survey administered in March 2021 was used to determine student awareness and use of the campus food pantry and reasons for not using the pantry (*n* = 888). When the survey was administered, campus activities and classes were held both in person and remotely with most classes being held fully remotely or offered as a hybrid. More information on survey administration is published elsewhere [26]. If students were aware of the pantry, they were asked whether they had utilized the pantry. If they had, they were asked about their satisfaction (5-point Likert scale, 1 being very dissatisfied and 5 being very satisfied). For pantry non-users, the survey assessed the rationale for not using the pantry, which included not needing it, feeling uncomfortable, inconvenient hours, inconvenient location, or other. Sociodemographic characteristics collected include age, gender, college classification, race, country of birth, first-generation student status, living situation, financial support, and meal plan status.

### 2.3. Student Satisfaction Survey

The standard campus recreation student satisfaction survey was modified to add additional questions on acceptability and satisfaction. Acceptability and satisfaction are defined as perceptions among consumers that a given program is agreeable, palatable, or satisfactory [27,28]. The added questions were adapted from validated survey measures [29,30] and two questions related to student satisfaction with the amount and variety of pantry foods available were created (see Supplementary Table S1). The two questions were pilot tested with undergraduate and graduate students before being added to the survey. Open-response questions were provided if students were unsatisfied or found the pantry unacceptable. After each pantry use, students were asked to fill out a student satisfaction survey regardless of if they had filled it out previously. The survey was anonymous and administered via Qualtrics, LLC (Provo, UT, USA). Students accessed the survey by scanning a QR code on their mobile devices as they left the pantry.

### 2.4. Statistical Analysis

Reach as an implementation science metric is calculated as the proportion of intended users that used the program [31]. Reach was calculated with intended users being the number of food-insecure students living on or near campus. This number was estimated during the first year of pantry operation using the percent of food insecure students estimated from the campus survey conducted in March 2021 (21.8%) [26] and the number of students that participated in campus-wide, mandatory COVID-19 testing during Spring 2021. Students who were attending the university remotely, due to the COVID-19 pandemic were excluded from these estimates because they were not able to utilize the pantry. Reach was then approximated as the ratio of students who used the pantry during the first year of operation to the number of food-insecure students living on or near campus.

Reach as a component of the Reach, Effectiveness, Adoption, Implementation, and Maintenance (RE-AIM) framework also includes reasons individuals choose not to participate [32,33] and demographic characteristics of participants and non-participants [34]. Demographic variables like race/ethnicity, age, and gender were also included in analyses because certain populations of students tend to be at higher risk of food insecurity [1,35,36] and may use campus food assistance resources differently—therefore, including these variables ensures populations of students are not overlooked if underrepresented. Descriptive statistics were calculated, and chi-square tests were conducted to determine whether there were categorical associations between sociodemographic characteristics and food security status, awareness, use, satisfaction, reasons students did not use the pantry, year of use, and total visits each academic year. All analyses were conducted in STATA/MP 14.1 (StataCorp, LP, College Station, TX, USA).

## 3. Results

### 3.1. Campus Food Pantry Awareness and Use from Campus Survey

Sociodemographic characteristics of campus survey respondents by awareness and use of the food pantry are presented in Table 1 along with a comparison of the campus student body. Students who were aware of the pantry were more likely to be first-generation (*p* = 0.02) and less likely to be foreign-born (*p* = 0.04). Food insecure students (*p* < 0.001) (those facing episodic (*p* = 0.05) or persistent food insecurity (*p* < 0.001)), Hispanic/Latinx students (*p* = 0.007), and those receiving financial support from the government (*p* = 0.002) reported using the campus food pantry more than others.

Demographic characteristics of food insecure students that used the pantry versus those that did not use the pantry were compared (Supplementary Table S1). Food insecure students that report not using the pantry during the first year of pantry operation were associated with being graduate students (*p* = 0.032) and having financial support from family (*p* = 0.052). Food pantry use among food-insecure students was associated with receiving financial support from the government (*p* = 0.016), loans (*p* = 0.052), or scholarships (*p* = 0.040).

Table 2 shows the awareness, use, satisfaction, and barriers to use the campus food pantry by food security status. There was no difference in awareness of the campus food pantry by food security status. More food-insecure students used the pantry (28.6%) compared to food-secure students (5.7%, *p* < 0.001). The majority of students that used the pantry were satisfied or very satisfied (74.0%). Significantly more food secure students (91.7%) reported not using the pantry because the assistance was not needed compared to food-insecure students (53.3%, *p* < 0.001). Lastly, more food-insecure students reported not using the food pantry because they were uncomfortable (30.0%) compared to food-secure students (2.3%, *p* < 0.001).

### 3.2. Food Pantry Reach and Patterns of Student Use from the Link2Feed Data

During the 2020–2021 academic year, there were 860 total student visits by 225 unique student users during 63 pantry openings. The average number of student visits per opening was 13.7 and the average number of visits per student was 3.8. For students that used the pantry more than once, the average number of visits was 6.5. The upper range of visits during the first year of operation was 36 visits. Campus-wide, an estimated 7276 students were affected by food insecurity during the Spring 2021 semester. Therefore, the pantry reached 3.1% of its target population.

During the second year of implementation (2021–2022), there was a total of 1401 student visits by 354 unique student users during 57 pantry openings. The average number of student visits per opening was 24.6 and the average number of visits per student was 4.0. For students that used the pantry more than once, the average number of visits was 6.9. The upper range of visits during year 2 was 38 visits.

From year 1, 55 students were returning users in year 2, visiting on average 13.3 times over the course of two years. The upper range of visits for two years was 58 visits. For students that used the pantry more than once during each academic year, the average number of visits was 16.2. Sociodemographic characteristics of student users are presented in Table 3 with students grouped per year, as returning users, and by the number of visits per year.

Of the total student users, most were female (59.9%), Asian/Pacific Islander (35.9%), lived in a private rental (59.9%), employed (62.8%), and 22 years or older (53.2%). Only 19.3% self-identified as being impacted by COVID-19. From year 1 to year 2, there were significant changes in user demographics. The number of undergraduate and younger students decreased (*p* < 0.001) and the number of graduate and older students increased (*p* < 0.001). There were also fewer Hispanic/Latinx students (*p* < 0.001) and more Asian/Pacific Islander students (*p* < 0.001) who used the pantry in year 2 than in year 1. Lastly, fewer students were impacted by COVID-19 during the second year of implementation (*p* = 0.003).

**Figure 1:**
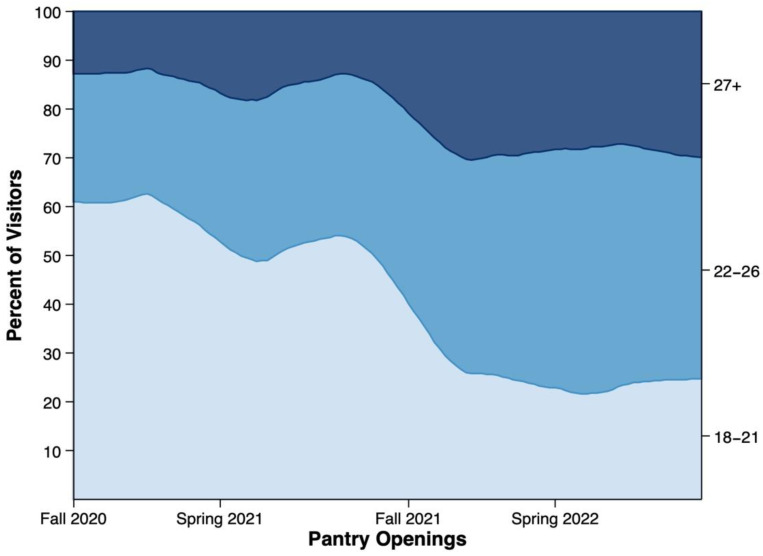
This graphic shows the percentage of students per opening from August 2020 through May 2022 with campus food pantry users grouped by age from Link2Feed data (*n* = 579).

Figure 1 shows the shift in students from year 1 to year 2 with students grouped by age. As shown in Figure 1, more than half of the pantry users during the first year of operation were younger students (aged 18 to 21 years). The percentage of pantry users aged 18 to 21 years began to drop toward the end of year 1 and continued until the middle of the following fall semester when this age group stayed around 25 to 30% of daily users. Nearly two-thirds of daily pantry users during the second year of implementation were older students aged 22+.

Referring to Table 3, about half (49.1%) of the students that used the pantry visited once (48.4% and 49.4% for years 1 and 2, respectively), 35.6% visited 2–7 times (37.3% and 34.5% for year 1 and 2, respectively), and 15.4% visited 8 or more times (14.2% and 16.1% for year 1 and 2, respectively). Students that were younger and undergraduate were associated with visiting the pantry fewer than 8 times (*p* < 0.05). Students who were employed were associated with visiting 2–7 times while older students and graduate students were associated with visiting 8 or more times (*p* < 0.05).

**Figure 2:**
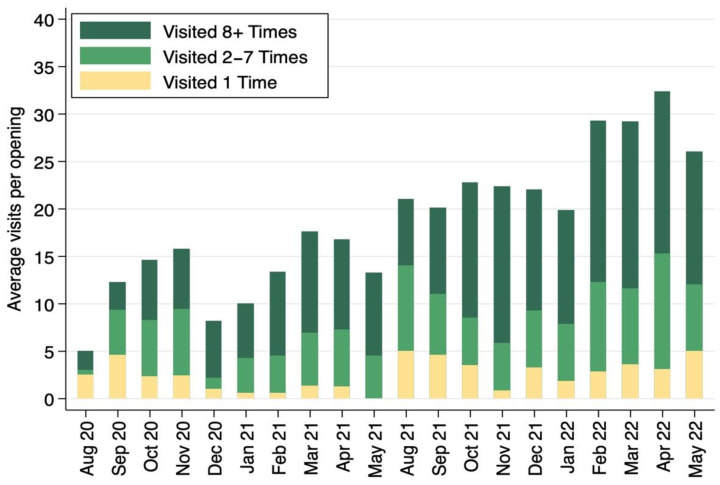
Average campus food pantry visits per opening per month from August 2020 to May 2022 with students grouped by yearly total visits; data from Link2Feed (*n* = 579).

The average visits per opening are graphed per month in Figure 2, with student users grouped by the number of visits during each academic year. In Figure 2, there is an overall increasing trend in the average visits per opening from August 2020 to May 2022 illustrating more students are using the pantry with time. There is a cyclical trend that occurs each semester with use peaking from the mid to the end of each semester. The average range of visits per opening was 5 to 32 with a large portion of daily visitors being those that visited eight or more times over the academic year.

**Figure 3:**
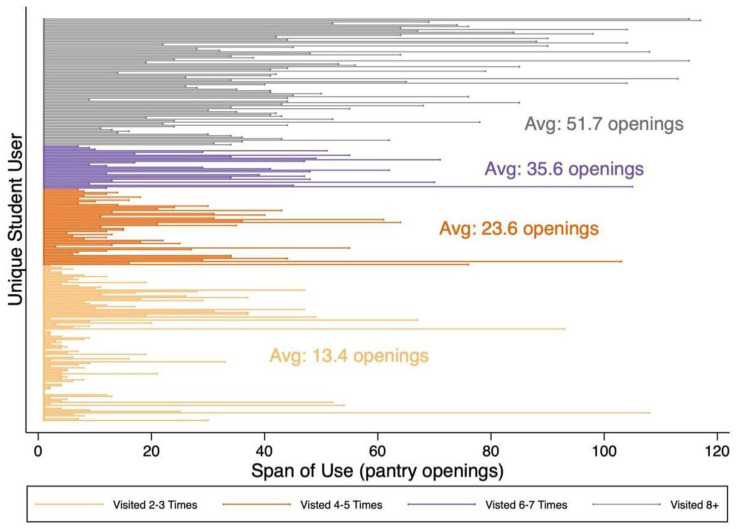
The span of use per student user with students grouped by the number of visits and the span is measured as the number of pantry openings between a student’s first and last visit. The average span of use is reported per group.

The span of use per student user is graphed in Figure 3, with students grouped by the number of visits and the span of use measured as the number of pantry openings between a student’s first and last visit. Figure 3 illustrates that on average, a student’s span of use increased as their total visits increased. Given the pantry was open twice a week, the average span of use for students that visited 2–3 times was 1.7 months, for those that visited 4–5 times the average span was 3.0 months, for those that visited 6–7 times the average span was 4.5 months, and those that visited 8 or more times the average span was 6.5 months. Students that used the pantry more also visited the pantry more frequently. The average number of openings between visits for those that visited 2–7 times ranged from 5.3–5.6, and for students that visited 8+ times, the average was 3.7.

Returning student users had a longer span of use compared to other students. Students that used the pantry during year 1 and year 2 that were not “returning users,” primarily used the pantry for 1 month (62.1%), and fewer students used the pantry for 2–4 months (27.9%), and 5–10 months (10.0%). For returning student users, only a third of students used the pantry for one month during each academic year (33.6%) and the majority used the pantry for 2–4 months (41.8%), with 24.6% using the pantry for 5–10 months each year.

Lastly, for patterns of monthly use, 75.8% of students visited once a month (76.0% and 75.7% for years 1 and 2, respectively), 16.6% visited twice per month (19.1% and 15.0% for years 1 and 2, respectively) and 7.6% visited more than twice a month (4.9% and 9.3% for year 1 and 2, respectively) during the months they used the pantry.

### 3.3. Acceptability and Satisfaction of the Campus Food Pantry from Student Satisfaction Surveys

Pantry survey results revealed students had high satisfaction and acceptability of the pantry. Out of 5 points, 5 being “very satisfied,” students, on average, rated satisfaction with food variety and healthy foods available at 4.5. Operating hours were rated slightly lower at an average of 4.3. For acceptability questions, average scores were between 4.8 and 4.9, with 5 being “strongly agree,” showing the highest amount of acceptability. Short answer questions revealed students want more fresh produce and spices/sauces available. The top reasons for dissatisfaction included expired food or produce nearing expiration and wanting extended hours of operation. Supplementary Table S2 includes the survey questions with mean scores per item and scale.

## 4. Discussion

This study assessed the reach, use, and satisfaction of a campus food pantry to inform the role campus pantries are playing in the food security safety net and improve pantry implementation. Our findings indicate most students used the campus food pantry in the short-term: more than half of students used the pantry for one month of support (62.1%), 84.6% visited the pantry fewer than 8 times during the academic year, and the longest average span of use for those that visited fewer than 8 times was 4.5 months. Graduate and older students relied on the pantry more than others and these students also had a longer span of use and visited more frequently. During the two years of operation, the percentage of students that visited the pantry 8 or more times in one academic year grew from 14.2% to 16.1% and the number of students that visited the pantry more than twice a month during the months visited nearly doubled (4.9% to 9.3%). Lastly, students had high satisfaction and acceptability of the pantry, yet the minority expressing dissatisfaction provided an opportunity for pantry improvements like increasing the amount of produce stored in the refrigerator due to feedback about produce nearing expiration.

Food secure and insecure students were equally aware of the campus food pantry. International students, however, were less aware of the pantry, which could be because students from other countries may be less familiar with the term “pantry” or the concept of charitable food programs if they do not exist in their home country. First-generation students were more aware of the pantry, perhaps hearing about it through formal or informal networks of support, seeking out resources, or being exposed to food pantries before college. The reach was relatively low during the first year of operation, which is consistent with previous research that suggests rates of utilization are generally low [16,17]. One study found that among three universities, 47.7% of students were aware of the campus food pantry and only 7.7% used the pantry [17]. Given the limited reach of campus pantries, some studies have investigated barriers to use, with the primary barrier being stigma [16,23,24]. Consistent with prior studies on barriers to use, we found significantly more food-insecure students were uncomfortable using the pantry, and about half of food-insecure students reported they did not need to use the pantry. While we do not know specifically why food-insecure students felt they did not need the pantry, previous research indicates that students often feel there are others with greater need and therefore are hesitant to seek support [16,23,24]. Students facing food insecurity also tend to employ other coping strategies for food acquisition and food management like buying the cheapest food available or stretching food to last longer [26,37]. Moreover, students facing persistent food insecurity employ coping strategies more frequently and were found to use other food acquisition coping strategies to a greater extent [26].

The campus survey found students who reported higher rates of pantry participation were food insecure. Most food-insecure students had experienced persistent food insecurity as opposed to episodic food insecurity. Among food-insecure students aware of the campus pantry, students relying on non-familial sources of support (e.g., scholarships, loans, and government aid) were associated with pantry use relative to food-insecure students that received financial support from their families. Most student pantry users were also employed and from the campus survey, students that reported higher rates of pantry participation were Hispanic/Latinx and received financial support from the government. These findings are consistent with other studies that found most students that use campus food pantries are food insecure, racial or ethnic minorities, and rely on multiple sources of support [16,17,18,20,22,38]. Previous research has found food insecure students and students that use campus food pantries are often employed at higher rates [4,18,22] demonstrating the magnitude of the financial challenge that attending college often presents [39].

Graduate students and older students tended to visit the pantry more frequently (8 or more times). Higher use among graduate students could be a result of less parental support, which wanes with age, and greater financial responsibilities (debt, etc.). If graduate students are employed by the university or elsewhere, their student wages may be insufficient to cover both living and academic expenses. This gap may be especially large for those at higher risk for food insecurity such as students with children [40]. Lastly, female students tended to use the pantry more than their male counterparts, which is consistent with other studies [20,21,22]. In our study, male students were less aware of the pantry compared to female students, which could have contributed. Therefore, efforts to increase awareness should target male students and make it more socially acceptable for them to access the pantry [16,41] given food security status does not significantly differ across gender [42,43,44]. Additionally, male students generally require more calories and specific nutrients to meet dietary recommendations.

Significant changes in student demographics were detected over the two years of operation. The first-year pantry of operation was in 2020, during the height of the COVID-19 pandemic when most classes were online, and many students were attending the university remotely. Significantly fewer students during the second year reported being impacted by COVID-19 compared to year 1. From year 1 to year 2, the main change in user demographics was in age and student classification with a large decrease in younger and undergraduate students. The cause for this change is difficult to pinpoint, however, one plausible explanation is the university had emergency funds for graduate students that experienced financial burdens during the pandemic (year 1 of pantry operation). This extra funding may have temporarily addressed graduate student food insecurity. Lastly, there was a significant change in the race/ethnicity of pantry users from year 1 to year 2 with more Asian/Pacific Islander and fewer Hispanic/Latinx student users. This change could be due to more students returning to campus during the second year of pantry operation when most classes returned in-person.

During the two years, there was an overall increase in the use of the campus food pantry in both total visits and the number of users. This finding is supported by previous research that tracked four years of pantry operation [22]. It is unclear whether the cause of this increase in use is due to greater food insecurity, greater awareness of the pantry [22,45], or rising food prices that were a result of the pandemic [46,47]. A prior study also found the busiest months in pantry use tended to be midway to late in the semester, which is consistent with our findings. Research conducted on campus food insecurity that evaluated the rate at multiple time points during the academic year found rates of food insecurity increased at the end of each semester and were highest at the end of the academic year [48]—this corresponds with findings on campus food pantry use.

Students reported high satisfaction with the pantry. Satisfaction and acceptability scores ranged from 4.3–4.9 out of 5 for all questions. Satisfaction with the hours of operation was the lowest ranked survey item and consistent with other findings [21]. Students rated satisfaction with food variety and healthy foods available at 4.5 out of 5. Based on qualitative responses from those unsatisfied with the pantry food supply, students wanted more fresh produce, spices, and sauces. The desire for more fresh produce is consistent with other studies [21,22,49]. If campus pantries remain as the sole or main campus food assistance resource, the nutritional quality of pantry items needs to be prioritized given some students may rely on pantry items as their sole source of food [16]. This study found at least half of monthly users are those that consistently acquire food from the pantry, visiting 8 times or more per academic year. Campus food pantries are uniquely positioned to promote health equity by providing nutritious foods that promote well-being and prevent disease to promote nutrition security for food-insecure students [50,51,52]. Students also noted dissatisfaction with expired foods or produce nearing expiration. Following these responses, pantry staff started to utilize the refrigerator for more produce storage and hung-up educational posters on the difference between “best by” versus “use by” labels. Continuing to collect satisfaction information will be important for continued program improvements.

While this study provided more insight into student reach and patterns of campus food pantry use, there were limitations. Data from the student satisfaction survey were self-reported and prone to social desirability bias given students who need food assistance may have been hesitant to rate low satisfaction if they want the university to keep the resource available to students. The campus survey and student satisfaction survey were only collected during year 1 and therefore satisfaction, reach, and awareness was not able to be recalculated during year 2. The Link2Feed administrative data had a question on student education level which was misinterpreted by some students (e.g., students as young as 18 selected “4-year degree” as their highest level of education completed). For this reason, not all students could be classified as undergraduate or graduate students. Lastly, students were able to fill out the student satisfaction survey each time they visited the pantry meaning some responses may belong to the same student. Thus, responses may not represent all pantry clients equally.

## 5. Conclusions

This study sought to provide more information on the extent of support students are seeking from campus food pantries to provide more insight into the role campus pantries are playing in the food security safety net. Based on our findings, there is a sub-set of students relying on the food pantry for long-term use while the majority use the pantry in the short-term. Given food insecurity is a spectrum, it may be difficult for one campus resource (e.g., campus food pantries) to equitably meet the needs of all students. Future research is needed to identify campus nutrition security interventions that are appropriate for all students to move more students from food sufficiency to nutrition security. Students that are food insecure but not accessing food pantries may call for targeted interventions to spread awareness, decrease barriers to use, or other programs that minimize stigma may need to be developed to better support students.

Universities could consider more affordable meal plans by creating eligibility criteria for students based on financial needs that mirror those offered by the National School Lunch Program (i.e., free or reduced-price meals). While meal plan status alone may not address campus food insecurity, lower-priced meals may afford students more money to spend on supplemental food or other essential items. More affordable meal plans may also reach more students that typically opt-out or do not purchase plans due to the high price. Long-term changes to college student Supplemental Nutrition Assistance Program eligibility may also better support students facing chronic and substantial food insecurity. Another study on campus food insecurity found students are interested in learning more about budgeting, cooking, and meal preparation [18]. Our findings from the satisfaction survey also suggest students are potentially interested in cooking given their request for more sauces and spices. Universities should explore providing free cooking and food resource management classes to improve student food literacy and promote life-long, healthy food resource management habits. Universities could also consider other ways to help cut costs of higher education for students, such as adopting open education resources that would alleviate the cost of textbooks and other school supplies. Alleviating other costs associated with higher education using systems-level approaches may also allow students to have more money for essentials. Overall, there is a need for universities to consider other campus programs and policies that equip food-insecure students with sustainable ways to acquire and manage food.

More research is needed to understand how students are utilizing campus food pantries over the course of the year and ways that college campuses can promote nutrition security. Implementation science could further what is known about pantry implementation, outreach, and operation. This information would give more insight into how students are utilizing food pantries to inform pantry improvements and campus leadership on the need to expand resources and tailor operations.

## Figures and Tables

**Table 1:** Sociodemographic characteristics of students who participated in an online campus survey and were aware of and used the campus food pantry compared with the campus student body (*n* = 888).

Characteristic	Student Body (*n* = 50,310)	Total (*n* = 888)	Aware ^a^ (*n* = 183)	Used ^b^ (*n* = 20)
Food Security Status, *n* (%)	NR			
Food Secure		694 (78.2)	141 (77.0)	8 (40.0) ***
Food Insecure		194 (21.8)	42 (23.0)	12 (60.0) ***
Episodic		101 (11.4)	21 (11.5)	5 (25.0) *
Persistent		93 (10.5)	21 (11.5)	7 (25.0) ***
Classification, *n* (%)				
Undergraduate	31,453 (63.6)	706 (79.7)	151 (82.5)	20 (100)
Graduate	17,987 (35.8)	180 (20.3)	32 (17.5)	0 (0.0)
First Generation, *n* (%)	7745 (23.1)	226 (26.5)	60 (32.8) *	8 (42.0)
International Student, *n* (%)				
US Born	41,358 (82.2)	709 (79.8)	160 (88.4)	18 (94.7)
Foreign Born	8952 (17.8)	143 (16.1)	21 (11.6) *	1 (5.3)
Gender, *n* (%)				
Female	27,596 (54.9)	353 (39.8)	108 (59.7)	9 (47.4)
Male	22,651 (45.0)	478 (53.8)	66 (36.5)	7 (36.8)
Unknown	63 (0.1)	57 (6.4)	7 (3.9)	3 (15.8)
Race/Ethnicity, *n* (%)				
Asian/Pacific Islander	8368 (16.6)	230 (25.9)	40 (21.9)	2 (10.0)
Black	2828 (5.6)	44 (5.0)	11 (6.0)	2 (10.0)
Hispanic/Latinx	5625 (11.2)	99 (11.2)	27 (14.8)	6 (30.0) **
White	20,448 (40.6)	420 (47.3)	90 (49.2)	6 (30.0)
Unknown	4089 (8.1)	95 (10.7)	15 (8.2)	4 (20.0)
Housing, *n* (%)	NR			
At home with family		127 (14.9)	23 (12.7)	4 (21.1)
Greek Life		42 (4.9)	6 (3.3)	0 (0.0)
Dorm		307 (36.0)	66 (36.5)	7 (36.8)
Apartment/House with roommates or spouse		372 (43.6)	86 (47.5)	8 (42.1)
Age (years), *n* (%)				
≤21	20,686 (41.2)	659 (74.2)	139 (76.0)	19 (95.0)
22+	29,574 (58.8)	229 (25.8)	44 (24.0)	1 (5.0)
Financial Support, *n* (%)	NR			
Family		625 (70.4)	138 (75.0)	12 (60.0)
Employment		405 (45.6)	89 (49.0)	9 (45.0)
Government		193 (21.7)	50 (27.0)	10 (50.0) **
Scholarships		311 (35.0)	79 (43.0)	11 (55.0)
Loans		213 (24.0)	58 (32.0)	8 (40.0)
Other		8 (0.9)	1 (1.0)	1 (5.0)
Meal Plan, *n* (%)	NR			
Has meal plan		314 (37.0)	76 (42.0)	8 (42.1)

NR = Not Reported. Chi-squared test was not conducted for categories where *n* < 5. *** *p* < 0.001, ** *p* < 0.01, * *p* < 0.0.5. ^a^ Pearson chi-squared tests compared sociodemographic characteristics among students aware of the campus food pantry to those not aware. ^b^ Pearson chi-squared compared sociodemographic characteristics among students who used the pantry to those that did not. Note: Comparisons of the student body and total student users from the campus survey were published previously [26]. “Reprinted from, The Journal of Nutrition Education and Behavior, 54, 11, Ana Mitchell, Brenna Ellison, Meg Bruening, Persistent and Episodic Food Insecurity and Associated Coping Strategies Among College Students, 972-981, 2022, with permission from Elsevier”.

**Table 2:** Prevalence of awareness, use, satisfaction, and barriers to using the campus food pantry by food security status among college students from a campus survey conducted in March 2021 (*n* = 888).

	Total (*n* = 888)	Food Secure (*n* = 694)	Food Insecure (*n* = 194)	*p*-Value ^a^
Aware of the pantry, *n* (%)				0.576
Yes	183 (21.0)	141(20.9)	42 (22.8)	
No	675 (79.0)	533 (79.1)	142 (77.2)	
Used Pantry ^b^, *n* (%)				0.000
Yes	20 (11.0)	8 (5.7)	28.6 (12.0)	
No	162 (89.0)	132 (94.3)	30 (71.4)	
Satisfaction with pantry ^c^, *n* (%)				
Very Dissatisfied	1 (5.0)	0 (0.0)	1 (8.3)	-
Neutral	4 (21.0)	1 (14.3)	3 (25.0)	-
Satisfied & Very Satisfied	14 (74.0)	6 (85.7)	8 (66.7)	0.363
Reasons for not using pantry ^b,d^, *n* (%)				
Not Needed	137 (84.0)	121 (91.7)	16 (53.3)	0.000
Location	9 (6.0)	6 (4.5)	3 (10.0)	0.239
Uncomfortable	12 (7.0)	3 (2.3)	9 (30.0)	0.000
Other ^e^	4 (2.0)	2 (1.5)	2 (6.7)	0.101

^a^ Pearson’s chi-squared tests determined categorical associations by food security status with significance set at *p* < 0.05. Responses less than 5 were not included in the analysis. ^b^ Question displayed for students aware of the pantry existing. ^c^ Question displayed for students who utilized the pantry; eleven respondents were very satisfied with the pantry and three were satisfied. ^d^ No one reported inconvenient hours as a reason for not using the pantry. ^e^ Other includes three students not on campus and one who forgot about the pantry.

**Table 3:** Summary of demographic characteristics among students, by year, returning students, and by the number of campus food pantry visits per year from Link2Feed data (*n* = 579).

	Total	Year 1 ^a^	Year 2 ^a^	Returning	1 Visit ^b^	2–7 Visits ^b^	8+ Visits ^b^
Characteristics	(*n* = 579)	(*n* = 225)	(*n* = 354)	(*n* = 55)	(*n* = 284)	(*n* = 206)	(*n* = 89)
Level of Education, *n* (%) ^c^							
Undergraduate	307 (53.2)	161 (71.9) ^a^	146 (41.4)^b^	31 (57.4)	162 (57.5) ^a^	115 (55.8) ^a^	30 (33.7) ^b^
Graduate	106 (18.4)	25 (11.2) ^a^	81 (23.0) ^b^	7 (13.0)	39 (13.8) ^a^	43 (20.9) ^a^	24 (27.0) ^b^
Unknown	164 (28.4)	38 (17.0) ^a^	126(35.7) ^b^	16 (29.6)	81 (28.7) ^ab^	48 (23.3) ^a^	35 (39.3) ^b^
Gender, *n* (%)							
Female	347 (59.9)	140 (62.2)	207 (58.5)	36 (65.5)	175 (61.6)	126 (61.2)	46 (51.7)
Male	219 (37.8)	81 (36.0)	138 (39.0)	17 (31.0)	105 (37.0)	76 (36.9)	38 (42.7)
Unknown	13 (2.3)	4 (1.8)	9 (2.5)	2 (3.6)	4 (1.41)	4 (1.9)	5 (5.6)
Race/Ethnicity, *n* (%)							
Asian/Pacific Islander	208 (35.9)	44 (19.6) ^a^	164(46.3) ^b^	17 (30.9)	93 (32.8)	81 (39.3)	34 (38.2)
Black	48 (8.3)	25 (11.1) ^a^	23 (6.5) ^a^	3 (5.5)	30 (10.6)	15 (7.3)	3 (3.4)
Hispanic/Latinx	134 (23.1)	73 (32.4) ^a^	61 (17.2) ^b^	12 (21.8)	72 (25.4)	47 (22.8)	15 (16.9)
White	116 (20.0)	55 (24.4) ^a^	61 (17.2) ^a^	10 (18.2)	52 (18.3)	42 (20.4)	22 (24.7)
Other	30 (5.2)	16 (7.1) ^a^	14 (4.0) ^a^	6 (10.9)	15 (5.3)	11 (5.3)	4 (4.5)
Unknown	43 (7.4)	12 (5.3) ^a^	31 (8.8) ^a^	7 (12.7)	22 (7.8)	10 (4.9)	11 (12.4)
Housing, *n* (%)							
Private Rental	347 (59.9)	128 (56.9)	219 (61.9)	31 (56.4)	176 (62.0)	114 (55.3)	57 (64.0)
With family/friends	82 (14.2)	34 (15.1)	48 (13.6)	8 (14.6)	34 (12.0)	33 (16.0)	15 (16.9)
School Housing/Dormitory	66 (11.4)	29 (12.9)	37 (10.5)	11 (20.0)	29 (10.2)	28 (13.6)	9 (10.1)
Other	44 (7.6)	23 (10.2)	21 (5.9)	2 (3.6)	25 (8.8)	17 (8.3)	2 (2.3)
Unknown	40 (6.9)	11 (4.9)	29 (8.2)	3 (5.5)	20 (7.0)	14 (6.8)	6 (6.7)
Age, *n* (%)							
≤21	271(46.8)	151 (67.1) ^a^	120 (33.9) ^b^	31 (56.4)	151 (53.2) ^a^	95 (46.1) ^a^	25 (28.1) ^b^
22+	308 (53.2)	74 (32.9) ^a^	234 (66.1) ^b^	24 (43.6)	133 (46.8) ^a^	111 (53.9) ^a^	64 (71.9) ^b^
Employment, *n* (%)							
Employed	364 (62.9)	147 (65.3) ^a^	217 (61.3) ^a^	34 (61.8)	166 (58.5) ^a^	149 (72.3) ^b^	49 (55.1) ^a^
Not Employed	145 (25.0)	63 (28.0) ^a^	82 (23.1) ^a^	14 (25.5)	81 (28.5) ^a^	39 (18.9) ^b^	25 (28.1) ^a^
Unknown	70 (12.1)	15 (6.7) ^a^	55 (15.5) ^b^	7 (12.7)	37 (13.0) ^ab^	18 (8.7) ^a^	15 (16.9) ^b^
Self-Identity, *n* (%)							
Disability	9 (1.6)	2 (0.9)	7 (2.0)	1 (1.8)	4 (1.4)	3 (1.5)	2 (2.3)
Impacted by COVID-19	112(19.3)	58 (25.8) ^a^	54 (15.3) ^b^	14 (25.5)	58 (20.4)	40 (19.4)	14 (15.7)
None	32 (57.3)	123 (54.7) ^a^	209 (59.0) ^a^	28 (50.9)	158 (55.6)	123 (59.7)	51 (57.3)
Other	37 (6.4)	12 (5.3) ^a^	25 (7.1) ^a^	5 (9.1)	19 (6.7)	11 (5.3)	7 (7.9)
Unknown	89 (15.4)	30 (13.3) ^a^	59 (16.7) ^a^	7 (12.7)	45 (15.9)	29 (14.1)	15 (16.9)

^a,b^ Pearson’s chi-square tests determined categorical associations between year 1 and year 2 student user characteristics and between the number of pantry visits per year. Superscripts are used to denote when significant differences exist with significance set at *p* < 0.05. The same superscript letters indicate that column proportions are not significantly different. ^c^ Undergraduate students were classified as students who answered High school, GED, Post-secondary (some), and 18- to 21-year-olds who selected a 4-year degree; graduate students are classified as those who selected master’s degree and Ph.D.; unknown level of education were those who did not select their highest level of education and those who selected a 4-year degree and were over the age of 21.

## Data Availability

Restrictions apply to the availability of these data. The data do not belong to the authors. Requests for data access can be sent to the corresponding author, and she will forward the request to the appropriate parties.

## Supplementary Materials

The following are available online at https://www.mdpi.com/article/10.3390/nu14224876/s1, Table S1: Sociodemographic characteristics of food insecure students who reported using and not using the campus food pantry based on results from an online campus survey (*n* = 888). Table S2: Satisfaction items and acceptability scale reported for college students that used a campus food pantry from August 2020 to May 2021 (*n* = 267).
