# Supplementary material for: The Role of Campus Food Pantries in the Food Security Safety Net: On-Going or Emergency Use at a Midwest Campus Pantry
Source: Nutrients. 2022 Nov 18;14(22):4876. doi: 10.3390/nu14224876 (PMC9692735; doi:10.3390/nu14224876)
Supplement: Supplementary file 1 [file nutrients-14-04876-s001.zip › nutrients-1996411-supplementary.pdf]

Table S1. Sociodemographic characteristics of food insecure students who reported using and not using the campus food pantry based on results from an online campus survey (n=888).

Characteristic	Used (n=12)	Did Not Use (n=30)	p-Value [a]
Food Security Status, n (%)			0.495
Episodic	5 (41.7)	16 (53.3)	
Persistent	*7 (58.3)*	14 (46.7)	
Classification, n (%)			0.032
Undergraduate	12 (100.0)	9 (30.0)	
Graduate	0 (0.0)	21 (70.0)	
First Generation, n (%)	*6 (50.0)*	10 (33.3)	
International Student, n (%)			-
US Born	11 (91.7)	26 (86.7)	
Foreign Born	1 (8.3)	4 (13.3)	
Gender, n (%)			0.841
Female	5 (41.7)	15 (51.7)	
Male	4 (33.3)	14 (48.3)	
Unknown	3 (25.0)	1 (3.3)	
Race/Ethnicity, n (%)			-
Asian/Pacific Islander [d]	1 (8.3)	8 (26.7)	
Black	2 (16.7)	2 (6.67)	
Hispanic/Latinx	3 (25.0)	3 (10.0)	
White	3 (25.0)	16 (53.3)	
Unknown	1 (8.3)	1 (3.3)	
Housing, n (%)			-
At home with family	2 (16.7)	3 (10.0)	
Greek Life	0 (0.0)	4 (13.3)	
Dorm	3 (25.0)	8 (26.7)	
Apartment/House with roommates or spouse	7 (58.3)	15 (50.0)	
Age (years), n (%)			-
≤ 21	11 (91.7)	21 (70.0)	
22 +	1 (8.3)	9 (30.0)	
Financial Support, n (%)			
Family	6 (50.0)	24 (80.0)	0.052
Employment	6 (50.0)	20 (66.7)	0.315
Government	8 (66.7)	8 (26.7)	0.016
Scholarships	9 (75.0)	12 (40.0)	0.040
Loans	6 (50.0)	6 (20.0)	0.052
Other	1 (8.3)	0 (0.0)	-
Meal Plan, n (%)			0.083
Has meal plan	4 (33.3)	11 (36.7)	

[a] Pearson's chi-squared tests determined categorical associations by pantry use with significance set at P < 0.05. Responses less than 5 were not included in the analysis

Table S2. Satisfaction items and acceptability scale items were reported for college students that used a campus food pantry from August 2020 to May 2021 (n=267).

Questions	N	Mean (SD)
Satisfaction [a]		
How satisfied were you with the variety of foods available?	267	4.5 (0.76)
How satisfied were you with the amount of healthy foods available?	260	4.5 (0.75)
I received help promptly.	226	4.5 (0.94)
The operating hours are convenient for me.	230	4.3 (1.1)
Acceptability [b]	243	28.9 (2.0)
How easy was it to use the Food Assistance and Wellbeing Program?	246	4.8 (0.40)
How helpful to you was the Food Assistance and Wellbeing Program?	245	4.9 (0.41)
Was the amount of time it took to use the Food Assistance & Wellbeing Program acceptable?	246	4.8 (0.42)
How would you rate your overall satisfaction with the Food Assistance and Well-being Program?	245	4.8 (0.45)
How much did you enjoy using the Food Assistance and Wellbeing Program?	245	4.8 (0.49)
How easy was it to understand how to use the Food Assistance & Well-being Program?	246	4.8 (0.50)

[a] Rated on a 5-point Likert scale, 1 being very dissatisfied and 5 being very satisfied
[b] Rated on a 5-point Likert scale, 1 being strongly disagreed and 5 being strongly agreed
